# Effectiveness and safety of azvudine in older adults with mild and moderate COVID-19: a retrospective observational study

**DOI:** 10.1186/s12879-023-08944-z

**Published:** 2024-01-04

**Authors:** Zhiguo Zhou, He Zheng, Gui’e Xiao, Xiangping Xie, Jiaxi Rang, Danhong Peng

**Affiliations:** 1 Department of Respiratory and Critical Care Medicine, The Affiliated Changsha Hospital of Xiangya School of Medicine, Central South University, The First Hospital of Changsha, Changsha, Hunan China; 2 Department of Infectious Disease, The Central Hospital of Shaoyang, Shaoyang, Hunan China; 3 Department of Nursing, The First Hospital of Changsha, Changsha, Hunan China

**Keywords:** Azvudine, Nirmatrelvir/ritonavir, COVID-19, Older adults, Treatment effect

## Abstract

**Background:**

Azvudine has clinical benefits and acceptable safety against COVID-19, including in patients with comorbidities, but there is a lack of available data for its use in older adult patients. This study explored the effectiveness and safety of azvudine in older adults with mild or moderate COVID-19.

**Methods:**

This retrospective cohort study included patients aged ≥80 diagnosed with COVID-19 at the Central Hospital of Shaoyang between October and November 2022. According to the therapies they received, the eligible patients were divided into the azvudine, nirmatrelvir/ritonavir, and standard-of-care (SOC) groups. The outcomes were the proportion of patients progressing to severe COVID-19, time to nucleic acid negative conversion (NANC), and the 5-, 7-, 10-, and 14-day NANC rates from admission.

**Results:**

The study included 55 patients treated with azvudine (*n* = 14), nirmatrelvir/ritonavir (*n* = 18), and SOC (*n* = 23). The median time from symptom onset to NANC of the azvudine, nirmatrelvir/ritonavir, and SOC groups was 14 (range, 6–25), 15 (range, 11–24), and 19 (range, 18–23) days, respectively. The median time from treatment initiation to NANC of the azvudine and nirmatrelvir/ritonavir groups was 8 (range, 4–20) and 9 (range, 5–16) days, respectively. The median length of hospital stay in the three groups was 10.5 (range, 5–23), 13.5 (range, 10–21), and 17 (range, 10–23) days, respectively. No treatment-related adverse events or serious adverse events were reported.

**Conclusion:**

Azvudine showed satisfactory effectiveness and acceptable safety in older adults with mild or moderate COVID-19. Therefore, azvudine could be a treatment option for this special patient population.

## Background

COVID-19 is an acute respiratory disease caused by SARS-CoV-2, a novel coronavirus closely related to SARS-CoV. The global COVID-19 pandemic has infected > 766 million people (confirmed cases) and killed > 6.9 million individuals as of May 14, 2023 [1–3]. The strains of SARS-CoV-2 are continuously evolving and show trends toward weaker virulence but higher infectiousness [4]. For example, the proportion of the BQ and XBB subvariants of the Omicron strain is increasing, and they manifest immune evasion [5]. The complications of COVID-19 contributing to mortality include coagulopathy, neurologic complications, and multisystem inflammatory syndrome [6, 7]. Advanced age is a high-risk factor of poor prognosis and death from COVID-19 [8, 9]. Several comorbidities known to worsen the prognosis of COVID-19 are commonly found in older adults, including cancer, cardiovascular diseases, kidney diseases, liver diseases, pulmonary conditions, rheumatic diseases, immune disorders, and musculoskeletal diseases [10–13]. The burden of disease in older adults is significant, with multiple comorbidities, severe physical or psychological symptoms, exacerbation of ageism, poor quality of life, difficult access to services, and reduced physical function [14, 15].

Antiviral drugs are considered the most effective therapies for COVID-19 and are recommended by the World Health Organization guidelines [6, 16]. The nirmatrelvir/ritonavir combination (co-packaged oral tablets, Paxlovid®) was approved for the treatment of mild-to-moderate COVID-19 in patients ≥12 years old and ≥ 40 kg with positive SARS-CoV-2 diagnostic test and who are at high risk of developing severe illness, including hospitalization or death [17, 18]. Unfortunately, antiviral drugs are expensive and in short supply, making them inaccessible to patients in developing countries. Furthermore, the treatment options for older adults with COVID-19 are limited because antiviral drugs can interact with various comorbidities to increase the risk of adverse events and may lead to nephrotoxic adverse reactions in patients with decreased kidney function or chronic kidney diseases [19, 20].

Azvudine is the first double-target nucleoside drug developed in China and was approved for COVID-19 treatment. Azvudine inhibits the nucleoside reverse transcriptase and restores cytosine deaminase APOBEC3G expression [21]. Azvudine can improve the lung function of patients with mild or moderate COVID-19, maintain their vital signs, reduce treatment time, and accelerate virus elimination [22, 23]. According to the available evidence on COVID-19 antiviral drugs, azvudine could be a therapeutic option for COVID-19 patients with comorbidities, considering its clinical benefits and acceptable safety [24–26]. Nevertheless, the available data on the use of azvudine in older adults are limited.

Therefore, this study aimed to explore the effectiveness and safety of azvudine in older adults with mild or moderate COVID-19. The results could help the management of COVID-19 in the population of patients with an advanced age who display multiple comorbidities and a higher risk of poor COVID-19 outcomes.

## Methods

### Study design and patients

This retrospective cohort study included older adult patients diagnosed with mild or moderate COVID-19 infection at the Central Hospital of Shaoyang (Hunan Province, China) between October and November 2022. This study was approved by the Ethics Committee of the Central Hospital of Shaoyang (approval number: KY-2022-002-20). Informed consent was waived by the Ethics Committee of the Central Hospital of Shaoyang due to the retrospective study nature.

The inclusion criteria were 1) ≥80 years of age, 2) positive SARS-CoV-2 (cycle threshold [Ct] value < 35) tested by reverse transcription-polymerase chain reaction (RT-PCR), 3) diagnosed with mild or moderate COVID-19 virus infection according to the “Diagnosis and Treatment Protocol for Novel Coronavirus Pneumonia (Trial Version 9), and 4) received antiviral therapy with azvudine or nirmatrelvir/ritonavir, or only received standard-of-care (SOC) therapy. The exclusion criteria were 1) treatment started at ≥5 days after the onset of symptoms, 2) use of other antiviral agents, or 3) incomplete key clinical data, such as the nucleic acid negative conversion (NANC) time.

### Grouping and treatment

According to the therapies they received, the eligible patients were divided into the azvudine, nirmatrelvir/ritonavir, and SOC groups. The dosage of azvudine was 5 mg orally once daily (QD). The dosage of nirmatrelvir/ritonavir was 300 mg of nirmatrelvir in combination with 100 mg of ritonavir administered orally twice daily (BID). Azvudine and nirmatrelvir/ritonavir were given for 5 consecutive days, and the dosages could be adjusted according to the creatinine clearance rate if necessary. The SOC therapy included traditional Chinese medicine (TCM), Lianhua Qingwen granules (LHQW), and ibuprofen. Oxygen therapy was used when the patients suffered from hypoxia.

### Data collection and outcomes

Age, sex, COVID-19 severity, doses of COVID-19 vaccines, comorbidities (including cardiovascular and cerebrovascular diseases, chronic lung diseases, diabetes, chronic liver and kidney diseases, tumors, acquired immunodeficiency syndrome, immune diseases related to long-term use of glucocorticoid and immunosuppressive drugs, etc.), COVID-19 symptoms (fever, asthenia, diarrhea, cough, nasal obstruction/running nose, rigor, panting, abdominal pain, diarrhea, headache, dizziness, palpitation, chest distress, chest pain, dry/sore/itchy throat, sore muscle, nausea, vomiting, dyspnea, gasteremphraxis, gastralgia, hypogeusia/poor appetite, hyposmia, etc.), and the Ct values of COVID-19 nucleic acids were collected from the medical records. According to “Diagnosis and Treatment Protocol for Novel Coronavirus Pneumonia (Trial Version 9)”, COVID-19 was classified as mild and common types. The common type is equivalent to the European Standard for moderate COVID-19.

The outcomes were the percentage of patients with COVID-19 progressing to severe diseases, NANC times, 5-, 7-, 10-, and 14-day NANC rates from admission, and length of hospital stay (LOS). The NANC times included 1) the time from the first occurrence of symptoms to the first NANC, 2) the time from the first positive nucleic acids to the first NANC, and 3) the time from starting using antiviral drugs to the first NANC (not available in the SOC group).

The Ct values of SARS-CoV-2 nucleic acids in nasopharyngeal swabs were tested daily by RT-PCR. Ct values > 35 were considered negative. Patients were discharged after two continuous negative nucleic acid test results performed at an interval of > 24 h. Treatment-related adverse events (TRAEs) and serious adverse events (SAEs) during hospitalization were recorded, if any.

### Statistical analysis

The statistical analysis was performed using R 4.2.1 (The R Project for Statistical Computing, www.r-project.org). Only descriptive statistics were used in this study. Age was presented as mean ± standard deviation, and the number of symptoms, Ct values, and NANC time were presented as medians (ranges). The categorical data were presented as n (%). The Kaplan-Meier method was used to estimate the NANC times and LOS, and the medians and 95% confidence intervals (CIs) were calculated.

## Results

### Characteristics of the patients

A total of 55 patients were included: 14 were treated with azvudine, 18 with nirmatrelvir/ritonavir, and 23 with SOC. In the azvudine and nirmatrelvir/ritonavir groups, one (7.1%) and three (16.7%) patients had mild COVID-19, while 21 (91.3%) had mild COVID-19 in the SOC group. The other baseline characteristics of the patients were generally balanced among the three groups (Table 1).

**Table 1:** Characteristics of the patients

Characteristics	Azvudine (*n* = 14)	Nirmatrelvir/ritonavir (*n* = 18)	SOC (*n* = 23)
Age (years), mean ± SD	82.9 ± 2.81	84.2 ± 4.12	85.5 ± 4.40
Sex (male), n (%)	6 (42.9%)	5 (27.8%)	9 (39.1%)
Severity at admission, n (%)			
Mild	1 (7.1%)	3 (16.7%)	21 (91.3%)
Moderate	13 (92.9%)	15 (83.3%)	2 (8.7%)
COVID-19 vaccine (≥ 1 dose), n (%)	4 (28.6%)	8 (44.4%)	11 (47.8%)
Underlying diseases, n (%)	9 (64.3%)	13 (72.2%)	16 (69.6%)
Number of symptoms, median (range)	1 (0–4)	1 (0–5)	2 (1–4)
Ct value, median (range)	21.3 (18.9–34.5)	21.3 (14.9–32.3)	22.1 (15.2–34.3)
Missing, n (%)	3 (21.4%)	3 (16.7%)	1 (4.3%)

*SOC* standard of care, *SD* standard deviation

### Clinical outcomes

**Fig. 1:**
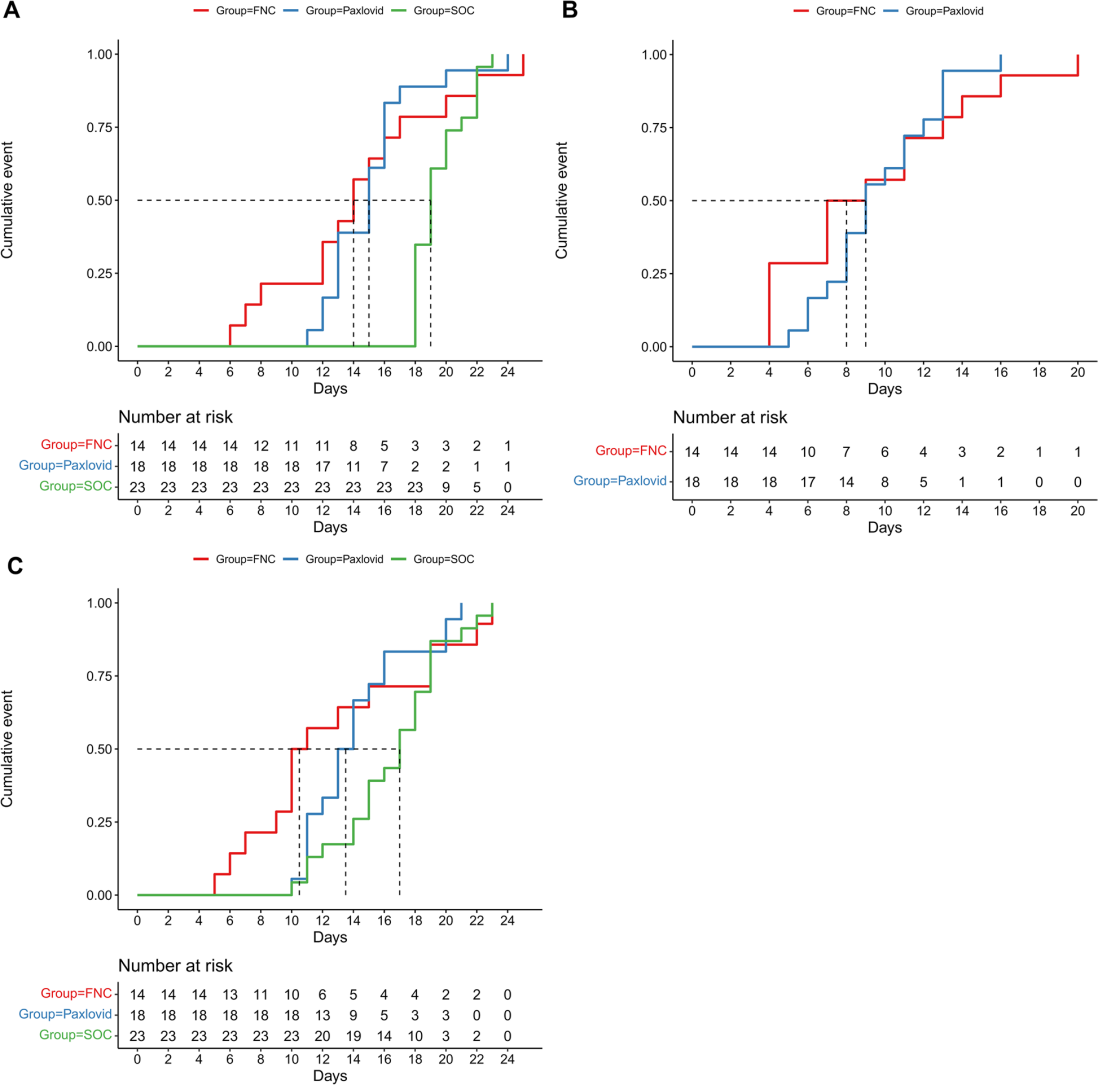
**a** Kaplan-Meier curves for the period from symptom onset to nucleic acid negative conversion (NANC). **b** Kaplan-Meier curves for the period from drug administration to NANC. **c** Kaplan-Meier curves for the length of stay

The median time from symptom onset to NANC of the azvudine, nirmatrelvir/ritonavir, and SOC groups was 14 (range, 6–25), 15 (range, 11–24), and 19 (range, 18–23) days, respectively. The median time from drug administration to NANC of the azvudine and nirmatrelvir/ritonavir groups was 8 (range, 4–20) and 9 (range, 5–16) days, respectively. The median LOS of the three groups was 10.5 (range, 5–23), 13.5 (range, 10–21), and 17 (range, 10–23) days, respectively (Fig. 1 and Table 2).

**Table 2:** Clinical outcomes

	Azvudine (*n* = 14)	Nirmatrelvir/ritonavir (*n* = 18)	SOC (*n* = 23)
Progressing to severe symptoms, n (%)	0	0	2 (8.7%)
NANC time (days), median (range)			
Symptom onset to NANC	14 (6–25)	15 (11–24)	19 (18–23)
Diagnosis to NANC	14 (6–25)	14.5 (11–24)	17 (10–22)
Hospital admission to NANC	9 (4–22)	12 (8–19)	15 (10–22)
Drug administration to NANC	8 (4–20)	9 (5–16)	NA
Length of stay	10.5 (5–23)	13.5 (10–21)	17 (10–23)
NANC rate from admission, % (95% CI)			
Within 5 days	14.3 (0.0,30.8)	0	0
Within 7 days	21.4 (0.0,40.2)	0	0
Within 10 days	57.1 (21.5,76.6)	27.8 (3.8,45.8)	17.4 (0.35,31.5)
Within 14 days	71.4 (34.6,87.5)	83.3 (53.2,94.0)	43.5 (19.1,60.5)

*SOC* standard of care, *CI* confidence interval

None of the patients on azvudine or nirmatrelvir/ritonavir progressed to severe COVID-19, while two (8.7%) in the SOC group progressed. The first NANC rates of the azvudine group within 5, 7, 10, and 14 days after hospital admission were 14.3, 21.4, 57.1, and 71.4%, respectively; those rates were 0, 0, 27.8, and 83.3%, respectively, in the nirmatrelvir/ritonavir group, and 0, 0, 17.4, and 43.5%, respectively, in the SOC group (Table 2).

The laboratory tests at admission and before discharge showed no clinically significant changes in liver and kidney functions in any patient. No TRAEs or SAEs were reported.

## Discussion

This study showed that azvudine could shorten the NANC time compared with standard symptomatic treatment in older adults with mild or moderate COVID-19. No patient receiving azvudine progressed to severe COVID-19. No TRAEs or SAEs during hospitalization were reported.

vaccines play a major role in the control of the COVID-19 pandemic [27, 28]. Still, immune drift and mutations are observed, and the new SARS-CoV-2 variants display immune evasion to the pre-existing immunity induced by vaccines or previous infections with other SARS-CoV-2 variants [29–32]. Vaccines can only be designed based on existing SARS-CoV-2 variants, and the next variants cannot be predicted with enough precision to design vaccines before their appearance [30], but immune imprinting will provide some protection against novel variants [33]. Therefore, vaccines will always lag compared with the circulating SARS-CoV-2 variants, and antiviral drugs still have their place in the armamentarium against COVID-19.

Azvudine is a nucleoside analog that is modified intracellularly to its active form that can inhibit viral RNA-dependent RNA polymerases [34, 35], inhibiting the replication of the hepatitis C virus, enterovirus 71 [34, 35], and the human immunodeficiency virus [36]. Recently, it was discovered that oral azvudine led to the accumulation of the active form in the thymus in rats, inhibiting SARS-CoV-2 replication, preserving thymus immune function, and rapidly curing COVID-19 [21]. It also restored the expression of cytosine deaminase APOBEC3G, which is a single-stranded DNA cytidine deaminase that targets retroviral minus-strand DNA, with protective effects against retroviruses [37].

In the present study, the proportion of patients with moderate COVID-19 was lower in the SOC group compared with the azvudine and nirmatrelvir/ritonavir groups (8.7% vs. 92.9% vs. 83.3%). It was consistent with clinical practice since physicians were more likely to administer antiviral drugs to patients with more severe diseases. Despite the higher proportions of patients with moderate COVID-19, the patients in the azvudine and nirmatrelvir/ritonavir groups had remarkably shorter time from symptom onset to NANC than the SOC group (median, 14 vs. 15 vs. 19 days). Approximately one-fifth of the patients receiving azvudine achieved NANC within 7 days, while no patients in the nirmatrelvir/ritonavir or SOC groups achieved NANC within 7 days. The NANC rate within 10 days from admission was numerically higher in the azvudine group than in the other two groups (57.1% vs. 27.8% vs. 17.4%). Although the NANC rate within 14 days in the azvudine group was slightly lower than in the nirmatrelvir/ritonavir group, it was higher than in the SOC group (71.4% vs. 83.3% vs. 43.5%). Therefore, azvudine could reduce the viral load and accelerate the clearance of the SARS-CoV compared with SOC. Several studies could show that azvudine can reduce the time to NANC and improve the symptoms of patients with COVID-19, including those with comorbidities [21, 22, 38, 39], as supported by a meta-analysis [40]. Azvudine also appears more effective than nirmatrelvir/ritonavir in patients with comorbidities [41]. Although those studies did not specifically include older adults, they exclusively examined azvudine in patients with comorbidities. Older adults usually have comorbidities such as frailty, malnutrition, and various chronic diseases.

Of note, two patients in the SOC group progressed to severe COVID-19, while no patients in the azvudine or nirmatrelvir/ritonavir group progressed. Sun et al. [26] showed that patients with comorbidities (e.g., chronic obstructive pulmonary disease and active cancer) treated with azvudine had a lower rate of progression to severe COVID-19 compared with the patients without azvudine but without differences in mortality. Besides, the azvudine group had shorter LOS than the nirmatrelvir/ritonavir and SOC groups (median, 10.5 vs. 13.5 vs. 17 days), indicating that azvudine may help save medical resources. No TRAEs, SAEs, or impairment in liver or kidney functions were reported with azvudine, suggesting good safety. Hence, azvudine could be a treatment option for mild and moderate COVID-19 in older adults.

Older adults represent a special population of patients with COVID-19. Indeed, frailty and malnutrition are often encountered in older adults and are factors of poor prognosis for many diseases, including COVID-19 [42, 43]. In addition, several comorbidities observed in older adults are factors of poor COVID-19 prognosis, including cancer, cardiovascular diseases, kidney diseases, liver diseases, pulmonary conditions, and rheumatic and musculoskeletal diseases [10–13]. Previous studies in older adults with COVID-19 showed that nirmatrelvir/ritonavir could improve the symptoms [44] and shorten the NANC time [45, 46]. A study in the USA supported the use of molnupiravir or nirmatrelvir/ritonavir in patients ≥65 years of age [47]. Similar results were observed in the present study, with nirmatrelvir/ritonavir shortening the NANC time compared with SOC. Still, the present study also showed that azvudine might be superior to nirmatrelvir/ritonavir with regard to the NANC time. Of course, considering the non-randomized design and small sample size, formal head-to-head clinical trials would be required to demonstrate the superiority. The shortening of the NANC time by azvudine has been shown in various patient populations, including patients with mild [23] and mild-to-moderate COVID-19 [22]. Both nirmatrelvir/ritonavir and azvudine are better than conventional SOC in terms of shortening the NANC time in patients ≥80 years of age, and no patients progressed to severe diseases, supporting the effectiveness of antiviral drugs in this special patient population.

A relatively high risk of drug-drug interaction has been reported with nirmatrelvir/ritonavir [48], which is worth noticing in older adults who are often receiving various medications for comorbidities. Nirmatrelvir/ritonavir is reported to be associated with TRAEs such as nausea, diarrhea, headache, running nose, and muscle ache [49], and several more severe TRAEs of nirmatrelvir/ritonavir (bradycardia and syncope) have been observed recently [50]. Few TRAEs of azvudine were previously reported [22], mainly headache, dizziness, increased aminotransferases, nausea, and increased D dimer levels [23]. No TRAEs were noted in the azvudine or nirmatrelvir/ritonavir groups in the present study, suggesting the acceptable safety of these antiviral drugs. Of note, both drugs were only used for 5 days, and follow-up for long-term safety is needed.

A previous study showed the effectiveness of nirmatrelvir/ritonavir in lowering the mortality of patients with severe COVID-19 [51]. Nevertheless, as the virus mutates and its lethality decreases, it has become a major concern to control the disease progression of patients with mild-to-moderate COVID-19 and reduce hospitalization. For older adults, prolonged LOS and long COVID-19 infection are important risk factors for low quality of life. This study preliminarily explored the effectiveness of azvudine in shortening the NANC times and the LOS, therefore providing certain guidance for clinical medication and directions for future studies.

This study has limitations. First, due to the small sample size, this study had low statistical power, and only descriptive analyses were performed. Second, as a retrospective study, there might be information bias, especially regarding TRAEs. In addition, the data were limited to those available in the medical charts. Third, because of the limited medical resources during the Omicron outbreak in late 2022 in China, the admission of some patients might have been delayed, and the time from diagnosis to NANC might have been lengthened.

## Conclusion

Azvudine showed satisfactory effectiveness and acceptable safety in older adults with mild or moderate COVID-19. Therefore, azvudine could be a treatment option for this special patient population.

## Data Availability

All data generated or analyzed during this study are included in this published article.

## Abbreviations

CIs: Confidence intervals
LOS: Length of hospital stay
LHQW: Lianhua Qingwen granules
NANC: Nucleic acid negative conversion
SAEs: Serious adverse events
SOC: Standard-of-care
TCM: Traditional Chinese medicine
TRAEs: Treatment-related adverse events
